# Breast Milk Lipidome Is Associated with Early Growth Trajectory in Preterm Infants

**DOI:** 10.3390/nu10020164

**Published:** 2018-01-31

**Authors:** Marie-Cécile Alexandre-Gouabau, Thomas Moyon, Véronique Cariou, Jean-Philippe Antignac, El Mostafa Qannari, Mikaël Croyal, Mohamed Soumah, Yann Guitton, Agnès David-Sochard, Hélène Billard, Arnaud Legrand, Cécile Boscher, Dominique Darmaun, Jean-Christophe Rozé, Clair-Yves Boquien

**Affiliations:** 1 Institut National de la Recherche Agronomique (INRA), Unité Mixte de Recherche (UMR) 1280, Physiopathologie des Adaptations Nutritionnelles, Institut des Maladies de L’appareil Digestif (IMAD), Centre de Recherche en Nutrition Humaine Ouest (CRNH), 44093 Nantes, CEDEX 1, France; Thomas.Moyon@univ-nantes.fr (T.M.); mikael.croyal@univ-nantes.fr (M.C.); agnes.david@univ-nantes.fr (A.D.-S.); helene.billard@univ-nantes.fr (H.B.); ddarmaun@gmail.com (D.D.); jeanchristophe.roze@chu-nantes.fr (J.-C.R.); clair-yves.boquien@univ-nantes.fr (C.-Y.B.); 2 Statistique, Sensométrie et Chimiométrie (StatSC), Ecole Nationale Vétérinaire, Agroalimentaire et de l’Alimentation Nantes-Atlantique (ONIRIS), Institut National de la Recherche Agronomique (INRA), 44322 Nantes, France; veronique.cariou@oniris-nantes.fr (V.C.); elmostafa.qannari@oniris-nantes.fr (E.M.Q.); molamine35@yahoo.fr (M.S.); 3 L’Université Nantes Angers Le Mans (LUNAM Université), Ecole Nationale Vétérinaire, Agroalimentaire et de l'Alimentation Nantes-Atlantique (ONIRIS), Laboratoire d’Etude des Résidus et Contaminants dans les Aliments (LABERCA), USC INRA 1329, 44200 Nantes, France; jean-philippe.antignac@oniris-nantes.fr (J.-P.A.); yann.guitton@oniris-nantes.fr (Y.G.); 4 Faculté de Médicine de Nantes, Centre Hospitalo-Universitaire Hôtel-Dieu (CHU), 44093 Nantes, France; arnaud.legrand@chu-nantes.fr (A.L.); cecile.boscher@chu-nantes.fr (C.B.); 5 European Milk Bank Association (EMBA), 20126 Milan, Italy

**Keywords:** breast milk lipidome, preterm infant, growth trajectory

## Abstract

Human milk is recommended for feeding preterm infants. The current pilot study aims to determine whether breast-milk lipidome had any impact on the early growth-pattern of preterm infants fed their own mother’s milk. A prospective-monocentric-observational birth-cohort was established, enrolling 138 preterm infants, who received their own mother’s breast-milk throughout hospital stay. All infants were ranked according to the change in weight *Z*-score between birth and hospital discharge. Then, we selected infants who experienced “slower” (*n* = 15, −1.54 ± 0.42 *Z*-score) or “faster” (*n* = 11, −0.48 ± 0.19 *Z*-score) growth; as expected, although groups did not differ regarding gestational age, birth weight *Z*-score was lower in the “faster-growth” group (0.56 ± 0.72 vs. −1.59 ± 0.96). Liquid chromatography–mass spectrometry lipidomic signatures combined with multivariate analyses made it possible to identify breast-milk lipid species that allowed clear-cut discrimination between groups. Validation of the selected biomarkers was performed using multidimensional statistical, false-discovery-rate and ROC (Receiver Operating Characteristic) tools. Breast-milk associated with faster growth contained more medium-chain saturated fatty acid and sphingomyelin, dihomo-γ-linolenic acid (DGLA)-containing phosphethanolamine, and less oleic acid-containing triglyceride and DGLA-oxylipin. The ability of such biomarkers to predict early-growth was validated in presence of confounding clinical factors but remains to be ascertained in larger cohort studies.

## 1. Introduction

A large body of epidemiologic evidence supports the short- and long-term benefit of breastfeeding in healthy term infants [1]. The World Health Organization (WHO) therefore recommends exclusive breastfeeding for the first six months of life [2]. Due to their tremendous growth rate, contrasting with the immaturity of their gastrointestinal system, most very preterm infants (born before 32 weeks of gestation) require intravenous nutrition for the first few weeks of life. As intravenous nutrition exposes to a high risk of sepsis, metabolic and hepatic complications, weaning preterm infants of intravenous nutrition is, however, a top priority and maternal milk feeding is strongly recommended in preterm infants as well [3,4]. When own mother’s milk is not available, the WHO and the American Academy of Pediatrics [5] recommend the use of donated breast milk, issued from a human milk bank, as an alternative [6,7,8]. In particular, feeding preterm infants with human milk has been shown to be associated with: (i) a dramatic reduction in the risk of developing prematurity-related morbidities [9], including necrotizing enterocolitis [10,11,12] and serious infection (sepsis) [7]; (ii) improved feeding tolerance and, in turn, faster advancement of enteral feeds [10,13]; and (iii) improved neurodevelopment [10,14]. Moreover, a few randomized controlled trials suggest breast milk might reduce the incidence of atherosclerosis in adolescents born preterm [15]. Breast milk alone, however, due to its relatively low protein and energy content, cannot cover the tremendous needs of preterm infants, and exclusive breastfeeding is often associated with extra-uterine growth restriction (EUGR) [14,16]. As impaired initial growth could have severe consequences since preterm birth per se is a risk factor for developmental delay [17,18], the European Society for Paediatric Gastroenterology Hepatology and Nutrition (EPSGHAN) committee on nutrition recommends human milk fortification for preterm infants [19]. Notwithstanding, a large range of variation is still observed regarding growth during hospital stay [20] even among preterm infants receiving protein-fortified human milk. Literature reports large inter-subject variations in the composition of expressed own mother’s milk [21]. Indeed, the fat composition can be directly impacted by numerous factors, including maternal diet [22,23], body mass index (BMI) [24], parity [25], or stage of lactation [26,27]. Regarding gestational age, it has been reported to impact total milk fat content only in the first stage of lactation [28,29]. Milk lipid supplies not only 45–55% of the total energy requirement of a healthy infant [30], but also building blocks for tissue growth. The effects of milk fatty acids and phospholipids on neurodevelopment have been widely studied [30,31,32]. However, there are scarce data on the potential health benefits of specific lipid components of human milk [30], specifically on preterm infants. Although metabolomics and lipidomics seem to be a promising approach in neonatology [33,34] to explore the broad range of concentrations of various components in small samples of biological materials [35], only a small number of clinical applications have appeared in literature [36,37,38]. Furthermore, to the best of our knowledge, no previous study has explored in depth the relationships between lipid components in human milk and the early growth of preterm infants during their hospital stay. To fill this gap, the current study aims at shedding light on the relationships between breast milk lipidome and growth pattern of preterm infants nourished by their own mother’s milk. In the present pilot study, we chose to select two groups of preterm infants, presenting very different growth trajectories during hospital stay, and selected within the ongoing LACTACOL birth cohort (ClinicalTrials.gov Identifier: NCT01493063, acronyme for the clinical study titled “Impact of Breast Milk on the Neurodevelopment of Preterm Newborns”). The aim of the present work therefore was: (i) to assess lipidome in the breast milk received by preterm infants during their first weeks of life; (ii) to evaluate the association between breast milk lipidome and the weight gain of infants between birth and time of discharge, by using multivariate statistical tools that accurately discriminate the two groups of preterm infants; and (iii) to identify a few breast milk lipid species and evaluate their predictive ability on the postnatal weight growth trajectory of the preterm infants, taking into account confounding clinical factors.

## 2. Materials and Methods

### 2.1. Study Design and Population

**Figure 1:**
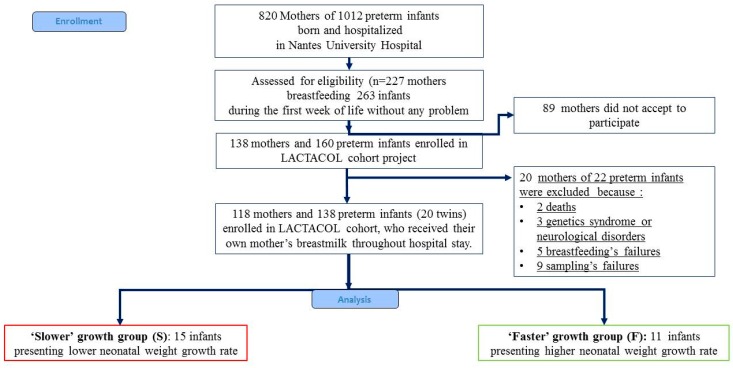
Flowchart of infants enrolled in the ancillary study of the mono-centric prospective population-based LACTACOL. (ClinicalTrials.gov Identifier: NCT01493063, acronyme for “Impact of Breast Milk on the Neurodevelopment of Preterm Newborns").

The present pilot study was an ancillary study conducted on sub-groups of infants selected among infants enrolled in the mono-centric prospective population-based LACTACOL birth cohort (registered at www.clinicaltrails.gov as NCT01493063) of preterm infant–mother dyads recruited from October 2011 to March 2016. The main objective of the LACTACOL cohort was to explore the impact of the protein content of breast milk provided to preterm infants during hospital stay on neurodevelopment at 2 years of age. A written consent was obtained from all participants at enrollment. One hundred thirty-eight infants born between 26 and 36 weeks of gestational age, with no major congenital disease except prematurity, were included, for a total of 118 mothers finally enrolled in the LACTACOL cohort (Figure 1). Necrotizing enterocolitis or retinopathy of prematurity were exclusion criteria. The development of comorbidities was defined as any of the following outcomes: intraventricular hemorrhage (IVH), defined as IVH associated with ventricular dilatation (grade III), cystic periventricular leukomalacia (cPVL) and ventricular dilatation, and/or severe bronchopulmonary dysplasia (BPD), defined as the administration of oxygen for at least 28 days plus the need for 30% or more oxygen and/or mechanical ventilator support or continuous positive airway pressure at 36 weeks’ postmenstrual age [39]. As reported in Table 1, none of the infants enrolled in the present pilot study presented any evidence of severe neonatal morbidity.

Infants admitted to the Neonatal Intensive Care Unit at Nantes University Hospital were eligible if they received human milk as their sole feeding for more than 28 days. Clinical characteristics were collected both on mothers and infants, including: maternal age, educational level, pre-gravid BMI, adverse events during pregnancy and delivery, infants’ gestational age, birth weight and head circumference, growth trajectory through hospital discharge, and events during hospital stay in neonatology. Parenteral nutritional supply was recorded daily, as well as enteral intake: volume of milk delivered per feeding session, fortifiers used and fortification level. The fortification followed, as far as possible, the EPSGHAN recommendations [19] regarding mean daily energy (i.e., 110–135 kcal/kg body weight/day) and macronutrient intake: 3.5–4.0 g/kg body weight/day for protein (for babies with 1–1.8 kg body weight); 4.8–6.6 g/kg body weight/day for lipid; and 11.6–13.2 g/kg body weight/day for carbohydrate. Human milk fortification was performed using Eoprotine^®^ (Milupa, Domdidier, Suisse) and FortiPré^®^ (Guigoz, Noisiel, France), for protein and carbohydrate; Liquigen^®^ (Nutricia, Saint-Ouen, France) for lipid; and Dextrin maltose, for carbohydrate. Energy and protein contents in preterm standard formula were 72 kcal/100 mL and 2 g/100 mL, respectively [40]. By taking into account native own mother’s milk macro-nutriments contents (measured by MIRIS human milk analyzer (Miris AB^®^, Uppsala, Sweden) analysis, Section 2.4) and routine fortification, the mean total enteral intake at 3 weeks of age, was: 2.48 ± 0.16 in the “faster growth” group, vs. 2.49 ± 0.12 g/kg/day in the “slower growth” group (*p*-value = 0.69) for protein; 6.76 ± 0.56 vs. 5.48 ± 0.45 g/kg/day (*p*-value = 0.08) for lipid; and 13.08 ± 0.59 vs. 14.14 ± 0.49 g/kg body weight/day (*p*-value = 0.31) for carbohydrate. The mean energy intake was: 125.8 ± 6.07 vs. 118.1 ± 4.99 kcal/kg body weight/day (*p*-value = 0.17). These values were reasonably close to that of EPSGHAN guidelines except for protein intake (which was 28% below recommended intake) whereas total energy intake was consistent with guidelines in both groups of infants. Preterm infants received parenteral nutrition and minimal enteral feeding, predominantly with expressed breast milk, for a minimum of two weeks.

### 2.2. Ranking Infants According to Early Growth Trajectory

Body weight was measured weekly from birth to discharge (accuracy of 0.1 g). Weight *Z*-score was calculated using the Lambda Mu Sigma (LMS) method [41] and Olsen’s preterm infant growth chart [42] was applied with respect to birth and discharge measurements. After completion of the cohort, for the specific purpose of the current pilot study, infants enrolled in LACTACOL cohort were ranked according to their growth trajectory during hospital stay. Weight gain during hospitalization was assessed as the change in weight *Z*-score (expressed in units of Standard Deviation (SD) using the SD of the term category as the benchmark) between birth and hospital discharge and all infants were then ranked, according to this difference in weight *Z*-score. We then selected two groups of infants who belonged either to the first or to the last terciles of growth rate in our population of 138 enrolled preterm babies (i.e., a difference between discharge and birth weight *Z*-score greater than −0.56 or lower than −1.06, respectively); those 2 groups of infants are termed infants with “faster growth” or “slower growth” in the following (Figure 1). The infants with “faster growth” (*n* = 11 infants (7 boys and 4 girls) presented a difference between discharge and birth weight *Z*-score of: −0.479 ± 0.189 (−0.668; −0.294) SD (median and 25% and 75% percentiles). The infants with “slower growth” (*n* = 15 infants (10 boys and 5 girls) presented a difference between discharge and birth weight *Z*-score of: −1.538 ± 0.417 (−1.953; −1.230) SD (median and 25% and 75% percentiles). Moreover, in the selection of the infants, we had two others constraints: the availability of sufficient own mother’s milk samplings from week 2 to week 7 of lactation, and of infant serum (results not shown in the present work). Two sets of twins belonged to the “slower” growth group and two others sets of twins followed opposite trajectories regarding their weight *Z*-score difference between birth and hospital discharge, i.e., one set of twins belonged to the “faster” growth group, and the other belonged to the “slower” growth group. Clinical characteristics of mother-infant dyads are summarized in Table 1. Although gestational age (30–31 weeks) and length of hospital stay (49–51 days) did not differ between the groups, the group of infants with “faster” growth had a 25% lower birth weight (i.e., 1.200 ± 0.293 kg vs. 1.605 ± 0.211 kg) combined with a 69% greater gain in weight *Z*-score between birth and discharge. This negative correlation between birth weight and weight *Z*-score at time of discharge has long been known, and was previously reported in the large Loire Infant Follow-up Team (LIFT) cohort of 2277 preterm infants by our team [40] and in another cohort [43].

### 2.3. Ethics

This research study, referenced BRD/11/02-Y at Nantes University Hospital, was approved, on 28 February 2011, by the National Data Protection Authority (Commission Nationale de l’Informatique et des Libertés, No. 8911009) and, on 19 July 2011, by the appropriate ethics Committee for the Protection of People Participating in Biomedical Research (CPP—Ouest I (Tours, France), reference CPP RCB-2011-AOO292-39). The LACTACOL cohort was registered at www.clinicaltrials.gov under # NCT01493063. The current data were obtained in the corresponding ancillary study N° 3 with the following outcome i.e., to assess the relationship between the composition of breast milk (lipidome in the present study) and preterm infant’s growth trajectory during the first few weeks of life. The milk biobank was approved by the Committee for the Protection of Persons in medical research (approval was granted 24 June 2010, reference CPP CB-2010-03). Parents received oral and written information in the maternity ward or neonatal unit, lactation support and training on proper sample collection from the study lactation consultant. A written consent was obtained from all parents at enrolment.

### 2.4. Human Milk Collection and Targeted Fatty Acid Analysis

Weekly breast milk expression was performed manually by mothers at home, using a Medela Manual Breast pump (Medela Inc., Etampes, France). Representative 24-h mature milk samples were obtained by pooling breast milk sampled from 5 to 6 bottles brought daily to the Milk Bank of the Nantes University Hospital. The whole milk pool was homogenized with a disruptor (Polytron, Lucerne, Switzerland) and kept frozen at −80 °C until analysis. Total milk fat was measured using the MIRIS^®^ human milk analyzer (Miris AB^®^, Uppsala, Sweden), based on mid-infrared methodology [44]. The modified liquid–liquid extraction method of Bligh-Dyer [45] was used to extract lipophilic metabolites. Two lipid analyses were achieved: a first targeted analysis of total fatty acids and a second untargeted lipidomic profiling. Total fatty acids were analyzed by gas chromatography using an Agilent Technologies 7890A^®^ instrument (Perkin Elmer, Villebon-sur-Yvette, France), following trans-esterification [46]. See Supplementary Materials online for detailed information about biochemical analysis and materials used.

### 2.5. Breast Milk Liquid Chromatography–High-Resolution-Mass Spectrometry (LC-HRMS)-Based Lipidomic Profiling

Following Bligh-Dyer extraction, the organic layers were dried and subsequently reconstituted in acetonitrile-isopropanol-water (ACN:IPA:H_2_O 65:30:5, *v*/*v*/*v*). A 1200 infinity series^®^ high performance liquid chromatography (HPLC) system (Agilent Technologies, Santa Clara, CA, USA) coupled to an Exactive Orbitrap^®^ mass spectrometer (Thermo Fisher Scientific, Bremen, Germany) equipped with a heated electrospray (H-ESI II) source (operating in polarity switch mode) was used for lipid profiling. The full instrument calibration was performed using a MSCAL6 ProteoMassT LTQ/FT-Hybrid ESI Pos/Neg^®^ (Sigma–Aldrich, St. Louis, MO, USA). Xcalibur 2.2^®^ (Thermo Fisher Scientific, San Jose, CA, USA) was used for data acquisition and analysis. Lipid species separation was performed on a reverse phase CSH^®^ C_18_ (100 × 2.1 mm^2^ i.d., 1.7 µm particle size) column (Waters Corporation, Milford, MA, USA) using ACN:H_2_O (60:40) and IPA:ACN:H_2_O (88:10:2) as solvent A and B, respectively; both containing 10 mM ammonium acetate and 0.1% acetic acid [47]. The precision associated with sample preparation and LC-HRMS measurement was determined based on a quality control (QC) consisting of a pool of 10 mothers’ milk provided by the milk bank of Nantes Hospital Center. Summary assay procedures have been detailed in Supplementary Material.

### 2.6. Data Analysis and Lipid Species Characterization

LC–ESI (positive/negative) HRMS raw data files were initially preprocessed with Xcalibur 2.2^®^ (Thermo Fisher Scientific, San Jose, CA, USA), converted to the (*.mzXML) cross-platform open file format, using MSConvert^®^ [48], and processed using Workflow4Metabolomics^®^ (W4M) (http://workflow4metabolomics.org) [49]. Lipidomic data were extracted using: (i) pre-processing with the open-source XCMS^®^ [50] within W4M [49], for nonlinear retention time alignment and for automatic integration and extraction of the peak intensities for each detected features (ions of given mass-to charge ratio and retention time [*m*/*z*; RT]), combined to CAMERA^®^ [51], for annotation of isotopes and adducts; and (ii) normalization of intra- and inter-batch effects by fitting linear or local polynomial regression models to QC samples [52]. The resulting XCMS [*m*/*z*; RT] features for each sample was subsequently manually sorted out according to their quality of integration and filtered by a 30% relative SD cutoff within the repeated pooled QC injections (see Dunn and co-workers’ recommendations [53]). Thereafter, accurate mass measurement of each putative metabolite was submitted to LIPID Metabolites and Pathways Strategy (LipidMaps^®^, www.lipidmaps.org) annotation. Moreover, the use of all ion fragmentation, when reverse phase chromatography was applied, helped us identify the proposed lipids by examination of the (pseudo) tandem mass spectrometry spectrum generated [47], combined with the use of an in-house reference databank [54].

### 2.7. Statistical Analyses

**Figure 2:**
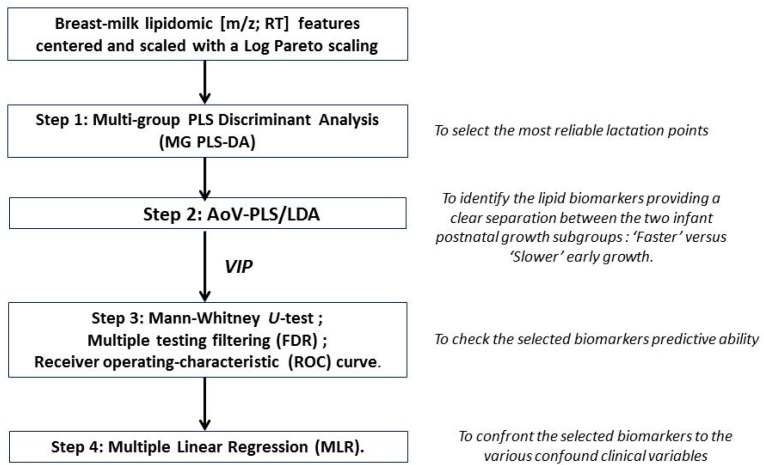
Statistical workflow applied on breast-milk Liquid Chromatography–High-Resolution-Mass Spectrometry profiles obtained in positive and negative ionization mode.

Statistical analyses were carried out using GraphPad Prism^®^ software version 6.00 (La Joya, CA, USA), SIMCA P^®^ version 13 (Umetrics AB, Malmö, Sweden) and R version 3.2.5 (R Development Core Team, 2013, R Foundation for Statistical Computing, Vienna, Austria; http://www.R-project.org). For all data analyses, the significance level (α) was set to 5%, and 10% for Multiple Linear Regression. Multivariate statistical models were applied on pre-processed QC-filtered lipidomic mother *x* time (rows) by [*m*/*z*; RT] features (columns) data matrix considering the a priori structure into “faster” vs. “slower” infants’ growth groups. Lipidomic features were column-wise centered and scaled with a Log Pareto scaling [55]. A statistical workflow (Figure 2) was set up to: (i) select the most reliable lactation points; (ii) identify the lipid biomarkers providing a clear separation between the two infant postnatal growth subgroups; (iii) check the selected biomarkers predictive ability; and (iv) confront them to the various confounding clinical variables.

Step 1: Selection of the most reliable lactation points. Multi-group PLS Discriminant Analysis (MG PLS-DA) [56] was performed to explain the a priori infant growth trajectory taking into account the longitudinal character of the data (weekly measurements) as groups among the rows (multigroup package in R) [57]. As a matter of fact, MG PLS-DA model revealed the time lactation points presenting the best differentiation between the two groups (“faster” vs. “slower” infant growth). Thereafter, only the most discriminant time lactation points were considered in the subsequent analyses and the significance of the growth factor was assessed. To cope with multi-colinearity inherent in metabolomics data [58,59], the Analysis of Variance-PLS (AoV-PLS) procedure proposed by El Ghaziri et al. [60] was applied.

Step 2: Identification of the lipid species biomarkers. AoV-PLS aimed at: (i) exploring how “faster” vs. “slower” preterm infants’ growth structure was explained by milk lipidomic signatures; and (ii) pinpointing the most discriminant features related to the infants’ growth pattern during hospital stay. This selection was performed on the basis of the variables of importance (for the milk clustering) indices (VIP > 1.0) [61] reflecting the contribution of each variable ([*m*/*z*; RT] feature) to the separation of the two infant groups. The appropriate number of components retained for the AoV-PLS model was chosen on the basis of the RMSEP (root mean square error of prediction) value obtained with a leave-one-out cross-validation procedure. In a subsequent stage, the selected AoV-PLS components were subjected to a Fisher’s linear discriminant analysis (LDA) to assess the significance of this model with respect with the two a priori groups (“faster” vs. “slower” infant growth) (function linDA of the DiscriMiner package in R [62]).

Step 3: Validation of the predictive ability of the selected biomarkers. To determine the more robust putative biomarkers of infant growth during hospital stay, lipid species that had been selected on the basis of AoV-PLS/LDA VIP indices in a multivariate scope were submitted to: (i) a subsequent univariate Mann–Whitney U-test; (ii) multiple testing filtering (FDR); and (iii) receiver operating-characteristic (ROC) curve (GraphPad Prism^®^). The parameter associated to the area under the curve (AUC) was set at 0.5 while the α-threshold was set at 0.05.

Step 4: Introduction of confounding clinical variables. Finally, confounding variables encompassing maternal and infant clinical data were introduced together with every selected putative biomarker to validate its reliability. The association between selected breast milk lipid species and child’s growth in terms of weight gain during hospital stay (delta *Z*-score) were investigated by means of Multiple Linear Regression (MLR), taking into account these confounding variables.

## 3. Results

### 3.1. A Distinct Breast Milk Lipidomic Signature Is Associated with Infant Growth Rate during Hospital Stay

Selected features of lipidomic LC-HRMS (ESI^+^/ESI^−^) profiles performed on breast milk samples from Week 2 to Week 7 of lactation were processed with MG PLS-DA (Step 1).

**Figure 3:**
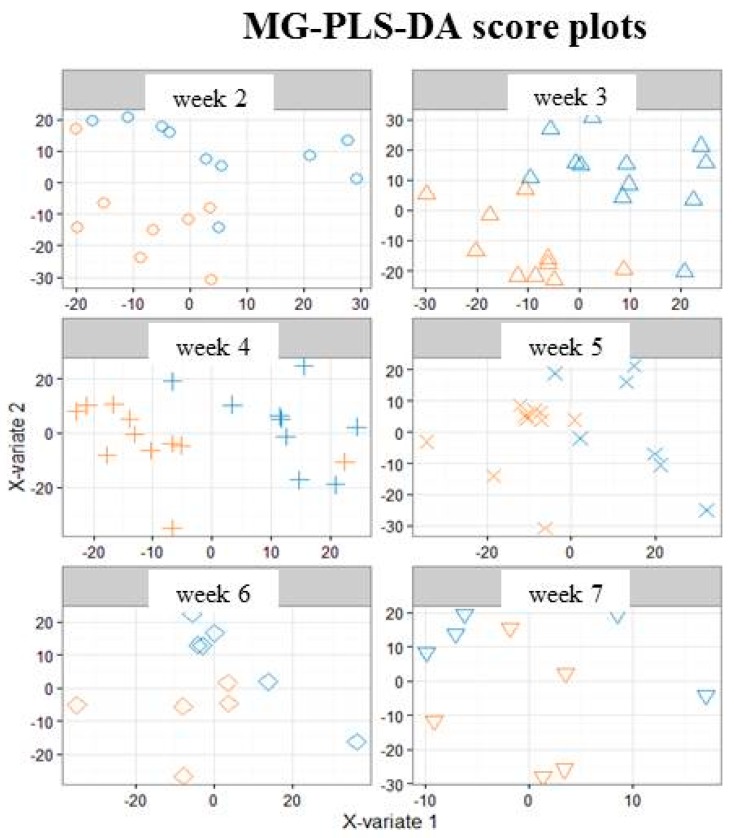
Multi-group PLS-DA score plots based on the LC-ESI^+^-HRMS profiles (3451 features, 118 milks) obtained on human preterm milk. Representation of the individuals: milk provided to preterm infants who experienced “faster” (orange) or “slower” (blue) growth from Week 2 to Week 7 of lactation. MB-PLS-DA score plots: ○ Week 2; ∆ Week 3; + Week 4; × Week 5; ◊ Week 6; ∇ Week 7.

On the corresponding score plots obtained in positive (Figure 3) and negative (Figure S1) ionization mode (3451 and 903 features respectively, 118 observations), the milk samples associated with the two groups (“faster” vs. “slower” growth) are plotted separately for each weekly sampling time point (i.e., Weeks 2 to 7). The first three sampling points at Weeks 2, 3 and 4 (i.e., from the 12th to the 24th day (median values)) allowed the best separation between the two groups of milks. This result led us to further restrict the multivariate analysis to data from samples obtained between Week 2 and Week 4.

**Figure 4:**
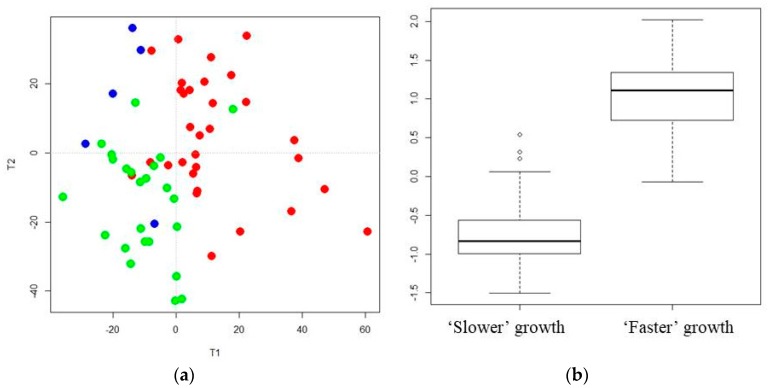
AoV-PLS and LDA models, based on the LC-ESI^+^-HRMS profiles of human preterm milk, on the factor weight *Z*-score (discharge-birth): AoV-PLS score plot with 45% of variance (R2Y = 38%) on: components 1–2 (**a**); and LDA (built on 10 components of AoV-PLS) with a *p*-value = 0) (**b**). Breast milk provided to preterm infants who experienced “faster” (green) or “slower” (red) growth and to twin infants with discordant growth rate, one with high growth rate and one with low growth rate, (blue).

AoV-PLS (Step 2) was applied separately on the LC-HRMS (ESI^+^/ESI^−^) to assess the association between the metabolites and the a priori grouping structure (“faster” vs. “slower” growth). The score plots clearly highlight the separation between breast milk lipidotypes associated with “faster” or “slower” infant growth in both positive (Figure 4a) and negative (Figure S2a) ionization modes. Interestingly, the breast milk lipidomic profiles, corresponding to two sets of twins with a concordant low growth rate, were plotted in the “slower growth” milk cluster. The lipidomic profiles of the breast milk provided to two sets of twins with discordant weight *Z*-score difference, one corresponding to “faster” (−0.668 and −0.479 SD) and the other to “slower” (−1.23 and −1.53 SD) growth, were found to be in an intermediate location between the two lipidotypes (depicted with blue symbols in Figure 4a and Figure S2). Then, the selected appropriate components of AoV-PLSs (for both ionization modes) were subjected to a Fisher’s linear discriminant analysis (LDA) to test the significance of growth factor.

The correlation ratio associated with the LDA canonical variable was equal to 71%, with respect to the positive mode (Figure 4b), and 61% with respect to the negative mode (Figure S2b), while their cross-validation error rates were both equal to 7.14%. The most discriminant features associated with infant growth during hospital stay corresponded to a cluster of 1006 (respectively, 256) VIP-based lipid species, in the positive (respectively, negative) ionization mode.

### 3.2. Characterization of Preterm Breast Milk Lipidotypes in the First Month of Lactation

Considering the targeted analysis of total fatty acids, milk provided to the “faster” growth group contained more total fat (4.75 g/100 mL) than the milk provided to infants with a slower growth rate (3.55 g/100 mL) from Week 2 to Week 4 (Table 2) (Table S1, for each sampling time, W2, W3 and W4 of lactation). This is, mainly due to a higher abundance of total saturated fatty acids (SAT) (free and triglycerides- and phospholipids-bound fatty acids) and particularly, medium-chain SAT (MCSAT). In contrast, total mono-unsaturated fatty acids (MUFA), the second most abundant class of milk fatty acids, were lower in the “faster growth” group than in the “slower” group, essentially due to lower oleic acid content. Milk provided to infants who experienced “faster growth” contained more overall n-3 long-chain PUFA, such as docosahexanoic acid and its precursors (eicosapentaenoic and docosapentaenoic acids). Finally, the essential FA content (i.e., linoleic and α-linolenic acids), was similar in both groups during this W2–W4 period.

Considering the untargeted lipidomic LC-HRMS signatures, among the 1262 features, selected as described above, 162 discriminant lipid species were annotated and are listed comprehensively in Table 3, for the overall W2–W4 lactation period, and in Table S2, for each sampling time, W2, W3 and W4 of lactation. Most of the annotated lipids were more abundant in the breast milk provided to infants with “faster” postnatal growth.

Indeed, these milks contained higher levels of medium- or long-chain sphingomyelins and ceramides [such as Cer (d18:1/24:0), SM(d18:0/12:0)], in several phosphatidylcholines, phosphatidylethanolamines or plasmalogen-derivatives containing palmitic (C16:0) or palmitoleic (C16:1) acid [such as PE (16:0/16:1), PE (16:1/20:0)], stearic (C18:0) or oleic (C18:1) acid [such as PC (18:0/18:1), PC (18:0/18:2) and PE (*O*-18:0/20:5)], dihomo-γ-linolenic acid (DGLA, C20:3) [PE (20:3/22:6), PE (22:0/20:3)], or docosahexaenoic acid (DHA, C22:6) [PE (20:3/22:6)]. In addition, breastmilk associated with “faster” growth contained less long-chain triglycerides (TG) containing oleic acid (C18:1) [such as TG (18:1/18:1/18:2), TG (16:0/17:1/18:1) and TG (18:0/18:1/18:1)]. Finally, several eicosanoids, including many DGLA-derived oxylipins [15S-HpEDE, 11-deoxy-16, 16-dimethyl- and 9-deoxy-9-methylene-16,16-dimethyl-PGE2], and Lyso-phospholipids (PL) [LysoPS (22:0) and LysoPG (22:4)] were lower in the breast milk provided to preterm infants with “faster” growth.

### 3.3. Reliability of Maternal Milk Lipids Biomarkers Regarding Postnatal Infant’s Growth

To assess the reliability of putative biomarkers as predictors of preterm infants’ growth during hospital stay, we considered the multiple testing filtering (i.e., adjusted FDR *p*-value < 0.05) (Step 3), combined, only for lipidomic LC-HRMS signatures, to the multivariate selection operated by the previously described AoV-PLS/LDA model. This approach led to the selection of four fatty acids [MCSAT and oleic acid (with a *p*-value of 0.051), EPA and DHA] and 46 robust biomarkers of infant postnatal growth, which were annotated and are reported in bold font in Table 2 and Table 3, respectively. In addition, the reliability of these selected biomarkers to predict postnatal growth rate was evaluated using a multiple linear regression (MLR) analysis (Step 4) to explain the change in weight *Z*-score between birth and hospital discharge. Several confounding clinical factors (mother’s body mass index, birth weight, gestational age, complementary parenteral and enteral nutrition with the protein, lipid and energy intakes, duration of parenteral feeding and ventilation and length of hospital-stay) were introduced in MLR models. The resulting MLR *p*-values of all these putative biomarkers are listed in Table 4.

**Figure 5:**
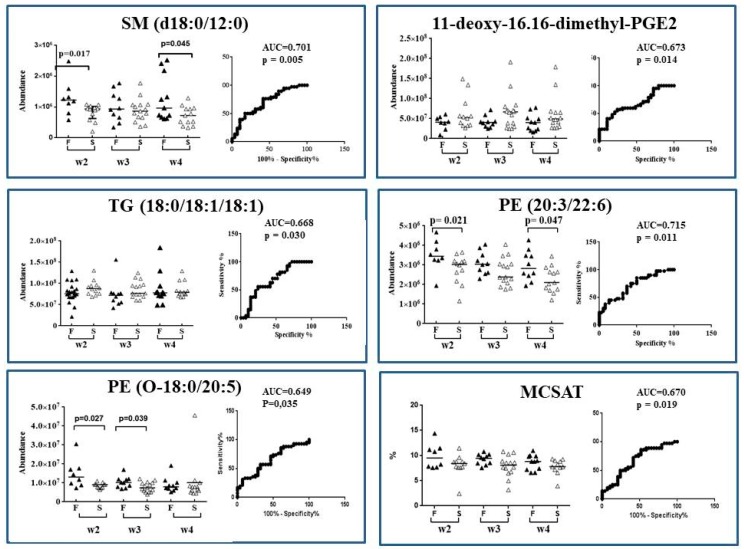
Scatter plot and ROC plot analysis using lipids biomarkers abundance (SM (d18:0/12:0), TG (18:0/18:1/18:1), PE (O-18:0/20:5), PE (20:3/22:6), MCSAT and one deoxy-dimethyl-PGE2) in breast milk provided to preterm infants who experienced “faster” (black triangle, F group) versus “slower” (white triangle, S group) growth during hospital stay. Scatter plot (median): from w2 to w4 of lactation period; ROC plot: over the entire W2–W4 lactation period. ▲ Faster growth group (F); Δ slower growth group (S).

Among the 50 AoV-PLS/LDA- and/or FDR-selected biomarkers, nine lipid species appeared of paramount interest, due to their significant (10%) MLR *p*-value for delta weight *Z*-score [two MCSAT, lauric (C12:0) and myristic (C14:0) acids; one MUFA, oleic (C18:1) acid; a medium-chain SM, SM (d18:0/12:0); a PE-plasmalogen containing stearic and EPA acid [PE (O-18:0/20:5)]; and two long-chain Lyso-PLs [LysoPS (22:0) and LysoPG (22:4)]; a PC-containing stearic and oleic acid, PC (18:0/18:1); a PE-containing DGLA and DHA, PE (20:3/22:6); DGLA-derived oxylipins [15S-HpEDE and two deoxy-dimethyl-PGE2]; and two TG-containing palmitoleic or oleic acid [TG (16:1/18:4/18:4), TG (16:0/17:1/18:1) and TG (18:0/18:1/18:1)]. The AUC of ROC curve of these nine lipid species ranged 0.6–0.7, indicating a reasonably good performance of these selected biomarkers to predict preterm infant weight growth during their first four postnatal weeks (as illustrated in Figure 5).

## 4. Discussion

To the best of our knowledge, the current pilot study is the first to demonstrate that clear-cut differences in breast milk lipidomic signature are associated with the early growth pattern of preterm infants receiving their own mother’s breast milk as their sole source of enteral feeding for the first month of life. Our strategy was to compare two groups of preterm infants who presented opposite growth velocity during their hospital stay. Infants in our “faster” growth group (mean birth weight *Z*-score −1.59 SD) lost less than 0.67 SD weight *Z*-scores between birth and discharge, indicating nearly optimal growth during their hospitalization. In contrast, infants in our “slower” growth group (mean birth weight *Z*-score 0.56 SD) lost more than −1.10 SD (range −1.953; −1.230) weight *Z*-scores between birth and discharge, indicating sub-optimal growth. These mean weight *Z*-score at discharge are nearly identical to the first and third tercile identified in a larger preterm cohort described earlier by our team [40]. A negative correlation between birth weight and early growth (between birth and hospital discharge) has long been known in cohorts of preterm infants [40,43]. We therefore introduced birth weight as one of the clinical parameters in the adjustment variables in Multiple Linear Regression (MLR) analysis. The latter model made it possible to check the reliability of potential biomarkers. In the following discussion, we focus on the biomarkers that remained significant after adjustment in MLR.

### 4.1. “Faster” Growth during Hospital Stay Is Associated with a Specific Maternal Milk Lipidomic Signature

We are aware of the fact that breast milk lipidomic signatures require ad hoc methods to assess the relevance of putative lipid biomarkers of preterm infant growth. For this purpose, we combined different multivariate statistical supervised models (MG PLS-DA, AoV-PLS followed to LDA), with various validation procedures [63]. These models allowed us to retrieve a set of 162 annotated lipids that contributed to breast-milk lipidotypes’ clustering associated to infants presenting “faster” or “slower” growth. The intermediate clustering of lipidotypes in AoV-PLS score plot for breast milk provided to the two sets of twins with discordant growth rates, suggests that postnatal growth rate is obviously multifactorial, and postnatal feeding is undoubtedly one of these factors. Stringency in our analysis was ensured by taking into account confounding clinical factors for the selection of biomarkers. Then, among the 50 FDR-selected lipids biomarkers, nine displayed good performance measures of infant’s weight gain during hospital stay, computed through a ROC curve [64], and a significant corrected MLR *p*-value for weight delta *Z*-score. The performance of ROC curve remained unchanged when discarding the two sets of twins who had discordant growth trajectories. This result suggests the reliability of the selected lipid species in the prediction of postnatal infant growth. These lipids present in breast milk therefore could be considered as robust candidate biomarkers of infant growth during hospital stay, in addition to classical determinants of postnatal growth of preterm infants during hospitalization such as birth weight, gestational age, development of comorbidities and protein/energy ratio in nutrition support [40]. It should be noted that the volume of milk intake, known to have a strong impact on early growth, was introduced in the Multiple Linear Regression model through the estimation of the total caloric intake. In the following, we therefore address the relevance of such putative MLR-significant biomarkers for preterm infant growth.

### 4.2. The Abundance of Mcsat in Breast Milk Is Associated with “Faster” Growth during Hospital Stay

Milk provided to preterm infants with “faster” growth contained more total MCSAT than milk provided to infants with “slower” growth. MCSAT may be important for the normal maturation of the gastrointestinal tract [65] and its protection from infection [66,67]. Moreover, MCSAT, and particularly lauric and myristic acids, are extensively oxidized [68]. Such an extensive oxidization leads to produce a dose-dependent rise in plasma ketones [69]. Ketones are a major source of both energy and acetyl-CoA not only for the brain development [69] but also for overall body growth. Such role of MCSAT could explain the association of MCSAT-rich milk with “faster” growth in our cohort.

### 4.3. Medium-Chain Sphingomyelins and Choline-Containing Phospholipids in Breast Milk Reliably Predict Early Growth in Preterm Infant

Infants with a faster growth received breast milk with a higher content in a medium-chain sphingomyelin SM (d18:0/12:0) and long chain choline-containing PLs:PC (18:0/18:1) and PE (20:3/22:6). In adults, medium chain-ceramides were found to enhance insulin sensitivity and improve glucose homeostasis [70,71]. Such an impact has not yet been explored in infants. Phosphatidylcholine and sphingomyelin were reported to protect against gastrointestinal infection, during early childhood, and to play a key role in gut barrier function [67]. With regard with phospholipids containing PUFAs, they may act as antioxidants in gut mucosa [72]. Finally, the abundance of phosphatidylcholine and sphingomyelin in breast milk implies that human milk supplies large amounts of choline, which is essential for neurodevelopment [32].

### 4.4. Enhanced Breast Milk Levels of DHA-, Dihomo-γ-Linolenic Acid- and Plasmalogen-Containing PE Are Associated with “Faster” Growth in Preterm Neonates

Breast milk provided to preterm infants with “faster” growth contained more DGLA- and DHA-containing PE (PE (20:3/22:6)). Such finding is consistent with the beneficial effects of DGLA reported in the treatment of inflammatory disorders and in cholesterol homeostasis [73]. Interestingly, our lipidomics study confirms the presence of plasmalogens from the PE (Pls-PEs) family. This has been previously reported in human milk [74] with a potential impact on infant health [66]. With respect with our data, the plasmalogen PE (O-18:0/20:5) containing EPA, a precursor of DHA, presented a significant MLR *p*-value corrected for both weight gain and head circumference growth in preterm infants. In contrast, breast milk total EPA and DHA content did not predict head growth in our infants, even though higher levels of EPA and DHA were found in the “faster” growth group. Taken together, these findings suggest dietary DHA, provided as a phospholipid may be more efficient than DHA supplied as part of a triglyceride, for brain DHA accretion, as reported in piglets [75]. Human milk plasmalogens may be involved in infant brain development since brain Pls-PEs were recently shown to accumulate postnatally and to be enriched in long-chain PUFA, particularly DHA [76]. Brain plasmalogen content was reported to increase between the 32nd week of gestation and the 4th and 6th postnatal month [66]. Plasmalogens exert an antioxidant effect [66]. The lower erythrocyte levels of plasmalogens reported in neonates, compared to older children, could be detrimental in preterm infants who have decreased anti-oxidant defense [77,78]. However, the biological determinants of Pls-PE FAs and physiological relevance to the breastfed infant remain to be elucidated [76].

### 4.5. Decreased Breast Milk Levels of Eicosanoids and Oleic Acid-Containing Triglycerides Are Associated with Early Weight Gain in Preterm Neonates

Decreased levels in long-chain TG containing oleic (TG (18:0/18:1/18:1) and TG (16:0/17:1/18:1)) predicted infant growth. Dietary substitution of medium-chain for long-chain triglycerides was shown to affect energy balance, promoting weight reduction in obese, adult humans [68]. In preterm infants, the use of lipid emulsions containing 50% medium-chain TG in parenteral nutrition was found to be associated with a lesser rate of protein accretion, compared to emulsions containing 100% long-chain TG [79]. Oleic acid may exert an anti-microbial activity and protect the digestive tract against infection [74]. However, it remains largely unexplored in infants [80]. Finally, among several eicosanoids that were less abundant in the breast milk provided to preterm infants’ with “faster” growth, three dihomo-γ-linolenic acid (DGLA)-derived oxylipins (15S-HpEDE and two deoxy-dimethyl-PGE2) displayed predictive ability on infant growth during hospital stay. These oxylipins are signaling lipids very recently found in human milk [73,81], and may play a role in preterm infant as pro- and/or anti-inflammatory mediators which could, in turn, exert key roles in maternal-infant biochemical imprinting [81]. Similarly, Lyso-phospholipids (LPLs) constitute another group of important lipid mediators with likely specific effects in infants that remained to be explored.

Taken in aggregate, the current findings suggest a striking inter-subject variation in the lipidome of breast milk among mothers who deliver preterm infants. Moreover, they suggest that such heterogeneity may impact early infant’s growth.

Limitations of the study stem from the relatively small size of the sample, the exploratory character of the study, and slight differences in birth weight between the groups. It has long been known that infants born with a lower birthweight grow faster, and this was previously reported in the large LIFT cohort of 2277 preterm infants by our team [40] as well as in another cohort [43]. Matching groups for birth weight would, therefore, be nearly impossible. We therefore have to admit that we cannot determine whether the difference in lipid species observed between groups in the current study reflects: (a) the difference in birth weight; (b) in growth trajectory per se; or (c) a combination of both. Delineating the relative contribution of the latter two factors (birth weight and early growth trajectory) and validating the ability of putative lipid biomarkers to predict early growth rate would require larger cohort studies. Moreover, our finding in twins, who followed opposite trajectories during their hospital stay, illustrates the fact that breast milk lipid composition is only one among the many factors that determine early growth in preterm infants.

The main strength of the study lies in the rigorous and comprehensive approach used to discriminate breast milk lipidomic patterns between the infant groups that experienced “faster” growth versus “slower” growth in their first four postnatal weeks. Robust lipid biomarkers, i.e., medium-chain sphingomyelin [SM (d18:0/12:0)], phospholipid containing DGLA and DHA [PE (20:3/22:6)], DGLA-derived oxylipin, TG-containing oleic acid (TG (16:0/17:1/18:1) and TG (18:0/18:1/18:1)) and MCSAT, displayed a good ability to predict weight gain during hospital stay, likely through their role in energy homeostasis, or the defense against oxidative stress, gastrointestinal tract infection, or inflammation. In particular, we confirm the presence of some new bioactive lipids (oxylipin) recently evidenced in human milk with suggested biological relevance to preterm infant health [82]. Further research on milk bioactive-lipid components is warranted to improve our understanding of the biological role of breast milk fat and its impact on infant development and health. Such understanding could, in turn, open the way to the manipulation of maternal diet to produce desired changes in breast milk, and/or to the use of specific lipid supplements in the personalized nutritional care of preterm infants in the neonatal intensive care unit.

## Figures and Tables

**Table 1:** Maternal and preterm infants’ characteristics.

	“Slower” Growth Rate	“Faster” Growth Rate	*p*-Value
**Maternal Characteristics**	11	11	
Age	30.00 ± 4.12 (26.00; 33.00)	29.00 ± 4.52 (25.00; 35.00)	0.908
BMI before gestation	24.00 ± 5.11 (20.83; 30.80)	22.32 ± 5.26 (19.14; 28.91)	0.789
**Infants characteristics at birth**	15 (10 males and 5 females)	11 (7 males and 4 females)	
Neonatal Morbidity (N° of events)			
Grade III IVH	0	0	
Cystic PVL	0	0	
Severe BPD	0	0	
Severe ROP	0	0	
Severe NEC	0	0	
Gestational age (w)	30.00 ± 1.68 (29.00; 32.00)	31.00 ± 1.37 (30.0; 32.00)	0.288
Hospital stay (d)	49.50 ± 4.21 (36.75; 54.75)	51.50 ± 3.16 (37.25; 56.25)	0.849
Birth weight (kg)	1.605 ± 0.211 (1.465; 1.705)	1.200 ± 0.293 (1.020; 1.445)	**0.005**
Birth length (cm)	41.20 ± 1.38 (39.00; 41.60)	38.00 ± 1.84 (37.00; 40.00)	**0.004**
Birth head circumference (cm)	28.00 ± 1.53 (27.00; 29.00)	26.20 ± 2.07 (25.505; 28.20)	0.202
Birth weight *Z*-score (SD)	0.564 ± 0.718 (−0.290; 0.842)	−1.592 ± 0.958 (−2.079; −0.571)	**0.000**
Birth length *Z*-score (SD)	0.210 ± 0.725 (−0.522; 0.746)	−0.793 ± 0.939 (−2.245; −0.129)	**0.003**
Birth head circumference *Z*-score (SD)	−0.036 ± 0.795 (−0.686; 0.386)	−1.528 ± 1.212 (−1.651; −0.128)	**0.015**
BMI at birth (kg/m^2^)	9.455 ± 0.857 (8.843; 9.900)	7.694 ± 1.573 (7.139; 9.884)	0.161
Discharge weight (kg)	2.565 ± 0.270 (2.355; 2.720)	2.340 ± 0.320 (2.029; 2.520)	**0.041**
Discharge length (cm)	45.00 ± 1.529 (44.10; 46.00)	44.00 ± 2.543 (41.00; 45.50)	0.219
Discharge head circumference (cm)	33.00 ± 1.412 (32.50; 34.00)	33.50 ± 1.511 (32.00; 34.00)	0.787
Discharge weight *Z*-score (SD)	−1.142 ± 0.682 (−1.552; −0.953)	−1.878 ± 0.857 (−2.264; −1.127)	0.146
Discharge length *Z*-score (SD)	−1.800 ± 0.713 (−2.242; −1.251)	−2.349 ± 1.054 (−2.803; −1.096)	0.466
Discharge head circumference Z-score (SD)	−0.681 ± 0.775 (−1.184; 0.301)	−0.216 ± 0.754 (−1.010; 0.1074)	0.655
BMI at Discharge (kg/m^2^)	12.67 ± 0.955 (11.78; 13.36)	11.98 ± 0.485 (11.66; 12.28)	**0.047**
Difference between discharge and birth weight *Z*-score (SD)	−1.538 ± 0.417 (−1.953; −1.230)	−0.479 ± 0.189 (−0.668; −0.294)	**<0.001**
Difference between discharge and birth length *Z*-score (SD)	−2.010 ± 0.752 (−2.474; −1.278)	−0.940 ± 0.723 (−1.822; −0.343)	**0.015**
Difference between discharge and birth head circumference *Z*-score (SD)	−0.113 ± 0.887 (−1.351; 0.258)	0.762 ± 1.122 (−0.248; 1.401)	**0.017**

Abbreviations: BPD, bronchopulmonary dysplasia; IPH, intraparenchymal hemorrhage; IVH, intraventricular hemorrhage; NEC, necrotizing enterocolitis; PVL, periventricular leukomalacia; ROP, retinopathy of prematurity. Severe bronchopulmonary dysplasia (BPD), defined as the administration of oxygen for at least 28 days plus the need for 30% or more oxygen and/or mechanical ventilator support or continuous positive airway pressure at 36 weeks’ postmenstrual age [39]. SD: standard deviation. Values are medians and 25% and 75% percentiles. *p* values for comparison between “faster” and “slower” growth groups were derived by using Mann–Whitney *U* test. Characteristics in bold font presented a corrected *p*-value < 0.05 between the two infant groups.

**Table 2:** Concentration levels of total fatty acids (free and triglycerides- and phospholipids-bound) in breast milk provided to preterm infants with “faster” or “slower” growth during the W2–W4 lactation period.

Fatty Acids (%)	W2 to W4		Mann–Whitney *p*-Value from W2 to W4	FDR Corrected *q*-Value from W2 to W4
	“Slower” Growth (*n* = 38)	“Faster” Growth (*n* = 29)		
8:0	0.176 (0.151–0.211)	0.198 (0.157–0.236)	0.201	0.183
10:0	1.654 (1.512–1.911)	1.791 (1.591–2.082)	0.101	0.132
12:0	6.203 (5.817–6.866)	7.100 (6.069–8.127) ^a^	**0.022**	***0.055***
**14:0**	7.049 (6.053–8.045)	8.039 (6.909–9.629) ^a^	**0.013**	**0.046**
16:0	23.19 (19.48–24.84)	23.15 (21.21–24.84)	0.677	0.353
18:0	6.872 (6.313–7.556)	6.934 (5.883–7.435)	0.406	0.279
20:0	0.195 (0.170–0.211)	0.196 (0.178–0.211)	0.872	0.379
**SAT**	45.74 (41.97–48.51)	48.08 (45.81–49.54) ^a^	**0.027**	***0.051***
**MCSAT**	8.050 (7.506–8.871)	8.976 (7.772–10.210) ^a^	**0.019**	***0.051***
16:1*_n_*_−9_	0.479 (0.419–0.530)	0.4573 (0.393–0.514)	0.192	0.183
16:1*_n_*_−7_	2.331 (2.030–2.737)	2.239 (2.098–2.698)	0.852	0.379
17:1*_n_*_−7_	0.222 (0.175–0.247)	0.228 (0.190–0.261)	0.310	0.249
18:1*_n_*_−9_	34.79 (32.25–37.88)	32.82 (31.02–34.78) ^a^	**0.027**	***0.055***
18:1*_n_*_−7_	1.720 (1.495–1.993)	1.818 (1.556–2.007)	0.468	0.287
20:1*_n_*_−9_	0.532 (0.483–0.567)	0.520 (0.462–0.597)	0.801	0.379
**MUFA**	41.10 (37.77–44.35)	39.58 (36.87–40.82) ^a^	**0.047**	***0.059***
**MUFA/SAT**	0.91 (0.79–1.04)	0.82 (0.74–0.91) ^a^	**0.037**	***0.059***
**18:1*_n_*_−9 a*n*d *n*−7_**	36.67 (34.09–39.79)	34.78 (32.76–36.61) ^a^	**0.028**	***0.051***
*cis* 18:2*_n_*_−6_ (LA)	9.651 (8.754–12.41)	8.881 (7.859–11.64)	***0.078***	0.117
*cis* 18:3*_n_*_−6_ (GLNA)	0.107 (0.086–0.133)	0.099 (0.078–0.125)	0.370	0.276
*cis* 20:2*_n_*_−6_	0.295 (0.252–0.332)	0.295 (0.235–0.343)	0.615	0.337
*cis* 20:3*_n_*_−6_ (DGLA)	0.349 (0.317–0.443)	0.404 (0.317–0.456)	0.429	0.279
*cis* 20:4*_n_*_−6_ (AA)	0.502 (0.425–0.580)	0.467 (0.379–0.591)	0.544	0.315
*cis* 22:2*_n_*_−6_	0.048 (0.040–0.061)	0.048 (0.039–0.060)	0.791	0.379
*cis* 22:4*_n_*_−6_	0.113 (0.091–0.135)	0.108 (0.086–0.134)	0.945	0.394
**Total n*-*6 PUFA**	11.44 (10.20–14.14)	10.38 (9.434–13.32)	***0.091***	***0.088***
*cis* 18:3*_n_*_−3_ (ALNA)	0.831 (0.609–1.140)	0.947 (0.720–1.285)	0.210	0.183
***cis*** **20:5*_n_*_−3_ (EPA)**	0.058 (0.042–0.077)	0.076 (0.061–0.092) ^b^	**0.006**	**0.046**
*cis* 22:5*_n_*_−3_ (DPA)	0.141 (0.114–0.172)	0.164 (0.140–0.180) ^a^	**0.057**	***0.098***
***cis*** **22:6*_n_*_−3_ (DHA)**	0.320 (0.220–0.390)	0.383 (0.316–0.477) ^a^	**0.013**	**0.046**
**Total n-3 PUFA**	1.598 (1.411–1.976)	1.881 (1.466–2.213) ^a^	***0.058***	***0.067***
**Total PUFA**	12.96 (11.74–16.40)	12.29 (11.15–15.65)	0.201	**0.159**
**Unsaturated/saturated fatty acid**	1.18 (1.06–1.38)	1.08 (1.02–1.18) ^a^	**0.023**	***0.052***
**PUFA/SFA**	0.29 (0.24–0.38)	0.26 (0.22–0.32)	0.101	***0.088***
**n-6/n-3 PUFA**	7.03 (5.83–8.23)	5.71 (5.04–6.87) ^2^	**0.005**	***0.052***
**LC-PUFA**	2.088 (1.768–2.321)	2.129 (1.978–2.383)	0.268	0.200
**Essential FA (LA + ALNA)**	10.73 (9.46–13.81)	9.83 (8.79–13.18)	0.101	***0.088***
**LA/ALA**	10.86 (9.30–15.66)	9.99 (8.35–11.69) ^a^	**0.047**	***0.059***
**AA/DHA**	1.679 (1.251–2.191)	1.307 (0.983–1.690) ^b^	**0.010**	***0.052***
**BCFA**	29.82 (28.65–30.99)	31.25 (29.99–32.51)	0.104	***0.088***
**Total lipids (Miris) (g/100 mL)**	3.55 (3.12–4.57)	4.75 (3.97–5.65) ^a^	**0.027**	***0.052***

PUFA: Polyunsaturated fatty acid; AA: arachidonic acid; EPA: eicosapentaenoic acid; DHA: docosahexanoic acid; DGLA: dihomo-gamma-linolenic acid; GLNA: gamma-linolenic acid; LA: Linoleic acid; ALNA: alpha-Linolenic acid; SAT: saturated fatty acids; MCSAT: medium-chain saturated fatty acids (C8:0 to C12:0); MUFA: monounsaturated fatty acid; LC-PUFA: long-Chain PUFA (polyunsaturated fatty acid that contains at least 20 carbons); BCFA: branched-chain fatty acids (C14:0, C15:0; C17:0 and C16:0). Values (expressed as % of total identified FA) are median and [25% and 75% percentile] and are given for fatty acids present at >0.05% of total fatty acids in milk. Values of *p*-values (assessed by Mann–Whitney *U* test) between “faster” and “slower” growth groups were reported with ^a^ or ^b^ significantly different, *p* < 0.05 or *p* < 0.01, respectively; *n*: milk samplings between Week 2 and Week 4 of lactation. Fatty acids in bold font presented a corrected *p*-value < 0.05 and fatty acids in bold and italic font presented a corrected *p*-value between 0.05 and 0.1, using the *post hoc* control of the type I error rate (False discovery Rate procedure), between the two infant groups over the entire W2–W4 lactation period.

**Table 3:** Abundance (10^6^) of annotated lipids that discriminated lipidotypes of breast milk provided to preterm infants with “faster” or “slower” growth during the W2–W4 lactation period.

Lipids	mz	Median [25% and 75% Percentile] from W2 to W4		Mann–Whitney U *p*-Value from W2 to W4	FDR Corrected *q*-Value from W2 to W4
		“Slower” Growth (*n* = 38)	“Faster” Growth (*n* = 29)		
**Fatty acid**		9.47 (7.40–12.84)	8.39 (5.53–13.00)	0.191	0.192
Anandamide (C18:3. n*-*6)	339.2889 [M + NH_4_]^+^	8.80 (7.02–11.77)	7.43 (5.11–11.47)	0.119	0.133
**3-Hydroxyadipic acid**	161.0455 [M − H]^−^	17.17 (14.02–20.25)	11.85 (5.68–15.09)	**0.000**	**0.000**
**N-formylmaleamic acid**	142.0203 [M − H]^−^	1.26 (0.84–1.64)	0.73 (0.59–0.97) ^c^	**0.000**	**0.000**
**Dodecatetraenedioic acid**	221.0667 [M − H]^−^	0.77 (0.53–0.88)	0.55 (0.34–0.73) ^b^	**0.000**	**0.001**
*Linderic acid*	187.1340 [M − H]^−^	0.52 (0.38–1.43)	0.76 (0.42–1.80)	0.282	***0.066***
*alpha-hydroxy lauric acid*	215.1653 [M − H]^−^	4.06 (2.39–8.23)	5.08 (2.51–13.03)	0.433	***0.087***
2-hydroxy palmitic acid	271.2281 [M − H]^−^	38.63 (22.48–48.63)	37.44 (29.98–50.32)	0.769	0.126
**3-oxo-4-pentenoic acid**	113.0243 [M − H]^−^	1.43 (1.18–1.71)	0.94 (0.69–1.28) ^c^	**0.000**	**0.000**
**Dehydrocholic acid**	401.2312 [M − H]^−^	0.43 (0.21–0.73)	0.52 (0.33–0.97)	0.211	**0.054**
*7R.9.14R-trimethyl-2E.4E.8E.10E-hexadecatetraenoic acid*	289.2169 [M − H]^−^	0.19 (0.10–0.48)	0.27 (0.14–0.46)	0.326	***0.072***
**Ceramide**		15.56 (12.98–18.63)	20.27 (17.34–23.79) ^c^	**0.000**	**0.000**
**Cer (18:1/22:0)**	622.6123 [M + H]^+^ 604.6017 [M + H-H_2_O]^+^ 644.5491 [M + Na]^+^	1.03 (0.89–1.38)	1.36 (1.14–1.69) ^b^	**0.005**	**0.032**
**Cer (d18:1/24:0)**	632.6326 [M − H_2_O]^+^	14.59 (12.11–17.50)	18.78 (16.21–22.15) ^c^	**0.000**	**0.011**
	650.643 [M + H]^+^				
**GlucosylCeramide**		511.4 (487.8–574.5)	519.5 (483.9–558.1)	0.764	0.385
Glucosylceramide (d18:2/14:0)	685.5361 [M + NH_4_]^+^	32.05 (13.43–46.52)	26.70 (13.73–41.90)	0.519	0.343
Galactosylceramide (d18:1/18:1)	743.614 [M + NH_4_]^+^	322.7 (309.6–355.8)	340.8 (316.5–348.9)	0.543	0.355
Galactosylceramide (d18:1/20:0)	773.652 [M + NH_4_]^+^	31.36 (29.58–33.78)	31.52 (30.08–33.87)	0.734	0.424
Glucosylceramide (d18:1/16:0)	717.5892 [M + NH_4_]^+^	42.06 (34.50–48.39)	42.43 (35.90–48.07)	0.9440	0.492
**Glucosylceramide (d18:1/18:0)**	728.5481 [M + H]^+^	2.28 (1.54–2.87)	1.85 (1.18–2.24) ^b^	**0.004**	**0.032**
Glucosylceramide (d18:1/20:0)	773.6614 [M + NH_4_]^+^	20.89 (19.20–22.66)	19.24 (15.94–22.67)	***0.092***	0.116
*Glucosylceramide (d18:1/24:0)*	854.7266 [M + Na]^+^	57.94 (54.82–62.99)	62.46 (56.55–66.47) ^t^	***0.068***	***0.099***
**Phosphocholine**		357.0 (315.6–456.2)	453.3 (370.1–568.0) ^b^	**0.006**	**0.009**
*PC (18:0/18:1)*	788.5863 [M + H]^+^	2.59 (1.55–4.34)	4.16 (3.06–5.25) ^a^	**0.025**	***0.064***
PC (14:0/16:0)	706.5391 [M + H]^+^	1.54 (1.09–2.63)	1.830 (1.47–3.3) ^t^	0.134	0.142
PC (14:0/16:1)	704.5237 [M + H]^+^	0.20 (0.09–0.40)	0.25 (0.13–0.50)	0.277	0.224
**PC (20:0/20:2)**	842.636 [M + H]^+^	1.49 (1.11–2.33)	2.33 (178–3.33) ^b^	**0.017**	**0.053**
PC (16:1/18:1)	1620.1146 [M + HPO_3_ + 2H]^+^	1.06 (0.89–1.21)	1.03 (0.89–1.14)	0.351	0.358
PC (16:1/18:0)	1542.1426 [2M + Na]+	11.76 (8.45–13.37)	10.58 (9.23–11.88)	0.387	0.277
*PC (16:1/18:2)*	1512.1349 [2M + H]+	2.33 (1.70–2.96)	3.23 (1.762–3.79) ^t^	**0.030**	***0.069***
PC (18:0/18:1)	1576.2229 [2M + H]+	12.09 (7.30–23.45)	16.68 (8.09–28.81)	0.150	0.153
*PC (18:1/18:1)*	1572.1908 [2M + H]+	103.2 (84.86–131.5)	127.6 (99.45–164.00) ^a^	**0.036**	***0.074***
**PC (18:0/18:2)**	786.5899 [M + H]^+^	7.02 (5.92–9.61)	10.60 (8.13–13.21) ^c^	**0.000**	**0.016**
**PC (16:0/20:3)**	784.5833 [M + H]^+^	51.58 (41.81–65.60)	65.96 (52.12–85.89) ^b^	**0.004**	**0.030**
PC (18:0/20:1)	816.6456 [M + H]^+^	11.92 (7.23–18.88)	12.22 (8.59–20.67)	0.386	0.277
**PC (18 :0/20 :3)**	812.6143 [M + H]^+^	30.80 (25.22–44.50)	46.59 (37.16–55.24) ^c^	**0.000**	**0.016**
PC (18 :0/20 :5)	808.5806 [M + H]^+^	93.34 (74.64–112.8)	94.55 (78.87–119.9)	0.343	0.256
**PC (20:1/20:4)**	836.6145 [M + H]^+^	3.72 (2.96–4.54)	4.88 (3.77–6.26) ^a^	**0.007**	**0.037**
PC (18:0/22:6)	834.596 [M + H]^+^	11.95 (8.65–15.21)	12.61 (10.11–17.45)	0.153	0.154
**PC (20:3/22:6)**	856.5807 [M + H]^+^	5.42 (3.95–6.97)	6.46 (5.06–7.62) ^a^	**0.013**	**0.047**
**PC (16:0/22:6)**	806.5678 [M + H]^+^	12.08 (7.25–16.08)	16.07 (12.04–24.40) ^a^	**0.007**	**0.037**
**PC-plasmalogen**		8.16 (6.53–10.52)	8.88 (6.97–12.23)	0.273	0.236
**PC (P-18:0/18:0)**	796.6199 [M + Na]^+^	1.72 (1.19–2.49)	2.32 (1.85–3.09) ^b^	**0.008**	**0.037**
PC (P-16:0/18:2)	742.5723 [M + H]^+^	2.18 (1.61–2.98)	1.59 (1.09–2.52] ^t^	0.104	0.124
PC (O-16:0/18:1)	768.5514 [M + H]+	4.11 (3.36–5.58)	4.82 (3.42–6.81)	0.114	0.131
**Phosphoethanolamine**		279.4(253.2–317.5)	316.5 (283.1–356.3) ^b^	**0.002**	**0.004**
**PE (16:0/16:1)**	690.5054 [M + H]^+^	1.79 (1.59–2.22)	2.15 (1.93–2.45) ^b^	**0.005**	**0.032**
*PE (16:1/20:0)*	1492.1294 [2M + H]+	6.72 (5.45–8.88)	8.38 (6.85–10.43) ^a^	**0.037**	***0.074***
*PE (16:0/20:2)*	1488.0975 [2M + H]+	21.16 (18.3–27.46)	27.62 (18.61–34.14) ^a^	**0.039**	***0.075***
**PE (16:0/20:2)**	744.5519 [M + H]^+^	142.2 (132.7–168.4)	166.6 (151.1–179.3) ^a^	**0.016**	**0.050**
*PE (16:0/20:4)*	779.5379 [M + Na]^+^	0.67 (0.57–0.87)	0.81 (0.61–1.19) ^a^	**0.044**	***0.079***
PE (18:0/20:0)	776.60 [M + H]^+^	1.68 (1.29–2.50)	1.52 (1.25–1.73) ^t^	0.131	0.141
*PE (20:1/20:4)*	794.5676 [M + H]^+^	7.45 (6.07–10.05)	9.41 (8.22–10.29) ^a^	**0.030**	***0.069***
**PE (18:0/20:4)**	1536.0968 [2M + H]+	4.93 (3.62–7.23)	7.71 (5.28–9.57) ^b^	**0.004**	**0.030**
**PE (22:0/20:3)**	826.6047 [M + H]^+^	2.09 (1.53–2.61)	2.51 (2.18–3.48) ^b^	**0.006**	**0.033**
PE (18:1/18:2)	1484.067 [2M + H]+	2.79 (1.97–3.99)	3.14 (2.07–4.36)	0.665	0.398
PE (16:0/20:4)	740.5208 [M + H]^+^	7.75 (5.31–9.68)	7.18 (5.44–9.98)	0.468	0.321
**PE (18:2/18:2)**	740.5213 [M + H]^+^	4.76(3.58–5.70)	6.21 (4.92–7.41) ^b^	**0.006**	**0.035**
**PE (20:0/18:1)**	774.599 [M + H]^+^	6.92 (5.61–9.12)	9.24 (7.24–11.69) ^b^	**0.004**	**0.030**
PE (18:0/20:4)	768.5495 [M + H]^+^	13.52 (12.42–14.25)	14.14 (13.13–15.75) ^t^	***0.094***	0.118
**PE (20:4/20:0)**	796.5836 [M + H]^+^	6.13 (4.98–8.72)	8.63 (6.81–10.52) ^b^	**0.007**	**0.035**
PE (22:6/18:0)	792.5517 [M + H]^+^	17.55 (12.31–22.26)	20.02 (15.51–25.01)	***0.085***	0.112
**PE (20:3/22:6)**	814.5336 [M + H]^+^	2.63 (1.96–3.06)	3.14 (2.58–3.59) ^c^	**0.002**	**0.023**
PE (22:0/22:6)	848.6566 [M + H]^+^	4.69 (3.55–6.02)	4.25 (2.64–4.98)	0.131	0.141
**PE (18:0/20:3)**	770.5672 [M + H]^+^	12.72 (10.13–16.13)	17.21 (14.84–20.82) ^c^	**0.000**	**0.001**
**PE-plasmalogen**		39.91 (31.47–49.92)	38.83 (34.83–52.40)	0.592	0.385
PE (*P*-16:0/20:5)	722.5102 [M + H]^+^	4.73 (3.96–7.45)	5.92 (4.46–7.69)	0.119	0.133
PE (P-16:0/18:0)	726.5323 [M + Na]^+^	10.82 (7.93–14.26)	9.44 (5.92–13.33)	0.186	0.173
PE (P-16:0/20:0)	754.5636 [M + Na]^+^	4.11 (2.58–5.23)	3.94 (2.25–4.96)	0.343	0.256
PE (P-16:0/20:3)	726.5417 [M + H]^+^	10.49 (8.45–12.70)	11.39 (9.41–14.01)	0.117	0.132
**PE (O-18:0/20:5)**	752.5551 [M + H]^+^	7.84 (6.04–9.98)	9.95 (7.30–12.09) ^a^	**0.019**	**0.056**
**Phosphatidylglycerol**		16.41 (15.59–18.36)	18.85 (16.90–22.25) ^b^	**0.002**	**0.004**
PG (16:0/16:0)	723.5101 [M + H]^+^	3.31 (2.77–3.90)	3.57 (2.85–3.79)	0.475	0.324
PG (18:0/20:4)	799.5425 [M + H]^+^	2.11 (1.72–2.36)	1.90 (1.48–2.25)	0.202	0.181
***PG (P-16:0/22:4)***	783.5586 [M + H]^+^	1.58 (1.26–1.76)	1.93 (1.47–2.26) ^a^	**0.021**	***0.058***
**PG (18:2/20:5)**	810.523 [M + NH_4_]^+^	6.52 (5.64–7.40)	7.67 (6.61–9.94) ^b^	**0.001**	**0.016**
*PG (22:2/22:6)*	892.5991 [M +NH_4_]^+^	3.20 (2.97–3.61)	3.56 (3.12–3.91) ^a^	**0.035**	***0.074***
**Phosphinositol**					
*PI (36:0) PI (18:0/18:0)*	889.5716 [M + Na]^+^	0.92 (0.78–1.39)	1.26 (0.89–2.20) ^a^	**0.055**	***0.087***
**Phosphoserine**					
**PS (40:5)**	838.552 [M + H]^+^	2.72 (2.52–3.06)	2.95 (2.64–3.11)	0.186	0.173
**PS (18:0/20:4)**	812.5419 [M + H]^+^	2.13 (1.59–2.77)	2.79 (2.29–3.26) ^b^	**0.002**	**0.023**
**Retinol**	287.2362 [M + H]^+^	2.02 (1.33–2.97)	1.57 (1.10–3.11)	0.752	0.429
**Diacylglyceride**		104.0 (66.98–163.6)	92.39 (52.86–126.5)	0.183	0.192
*DG (14:0/18:3)*	580.5373 [M + Na]^+^	8.52 (3.30–15.47)	4.92 (2.30–9.16) ^a^	**0.025**	***0.064***
DG (16:0/16:1)	567.4974 [M + H]^+^	6.61 (3.88–7.99)	5.26 (3.05–11.07)	0.656	0.395
DG 18:0/18:1)	645.544 [M + Na]^+^	7.56 (5.03–7.58)	6.90 (5.51–11.53)	0.797	0.444
*DG (18:0/18:2)*	638.5708 [M + NH_4_]^+^	55.02 (36.04–87.52)	40.60 (22.83–66.09) ^t^	**0.045**	***0.079***
DG (18:0/18:1)	634.5395 [M + NH_4_]^+^	10.80 (5.53–17.81)	10.46 (7.75–17.81)	0.582	0.370
DG (20:4/19:0)	681.5402 [M + Na]^+^	0.92 (0.47–1.60)	1.07 (0.60–2.03)	0.292	0.233
**DG (20:3:/20:0)**	*697.5727 [M + Na]^+^*	4.98 (2.91–9.25)	2.74 (1.64–4.81) ^a^	**0.027**	***0.065***
DG (18:3/20:0)	647.5574 [M + H]^+^	6.81 (2.59–13.46)	4.06 (1.51–9.17) ^t^	***0.079***	0.107
***DG (20:4/22:5)***	713.5103 [M + Na]^+^	0.56 (0.24–0.79)	0.26 (0.15–0.56) ^a^	**0.040**	***0.076***
**Triglyceride**		7758 (7304–8118)	7572 (7236–8496)	0.717	0.385
**TG (14:0/16:1/17:2)**	804.7056 [M + NH_4_]^+^	55.94 (47.00–60.12)	59.35 (52.54–69.66) ^a^	**0.011**	**0.043**
TG (14:0/14:1/14:1)	741.5983 [M + Na]^+^	415.0 (358.3–520.8)	415.2 (371.5–448.9)	0.972	0.499
**TG (14:0/16:0/16:0)**	796.7277 [M + NH_4_]^+^	337.3 (308.2–373.3)	381.5 (340.2–439.9) ^b^	**0.003**	**0.030**
TG (14:1/14:1/18:1)	790.6895 [M + NH_4_]^+^	1074 (923.6–1296)	1074 (911.2–1330)	0.788	0.442
**TG (14:0/14:1/19:1)**	806.712 [M + NH_4_]^+^	9.17 (7.68–10.01)	9.81 (8.58–10.81) ^a^	**0.012**	**0.046**
TG (13:0/14:1/20:5)	798.6639 [M + NH_4_]^+^	117.1 (114.0–124.0)	118.9 (111.6–124.5)	0.743	0.427
***TG (16:0/16:1/16:1)***	820.7365 [M + NH_4_]^+^	2033 (1831–2139)	2142 (1937–2447) ^a^	**0.028**	***0.067***
**TG (14:0/16:0/16:0)**	801.6948 [M + Na]^+^	25.93 (21.63–28.64)	29.18 (24.40–36.14) ^b^	**0.019**	***0.057***
TG (16:1/16:1/16:1)	823.676 [M + Na]^+^	257.9 (246.2–272.9)	258.8 (244.0–270.8)	0.639	0.388
TG (16:1/16:1/17:1)	832.7368 [M + NH_4_]^+^	74.98 (64.31–82.97)	76.59 (69.19–90.90)	0.178	0.169
TG (14:0/15:0/20:5)	828.7134 [M + NH_4_]^+^	10.48 (8.74–11.70)	11.27 (9.79–12.47)	0.178	0.169
TG (16:0/16:1/18:0)	850.7742 [M + NH_4_]^+^	320.2 (306.7–334.3)	325.7 (308.6–364.5)	***0.099***	0.121
*TG (16:0/17:1/18:1)*	862.8205 [M + NH_4_]^+^	3.37 (2.70–5.18)	2.93 (2.00–3.59) ^t^	**0.051**	***0.083***
*TG (16:0/16:0/18:1)*	850.7655 [M + NH_4_]^+^	12.01 (10.70–13.14)	11.06 (9.80–12.04)^1^	**0.021**	***0.058***
TG (16:1/16:1/17:2)	830.7291 [M + NH_4_]^+^	10.40 (9.81–10.85)	10.65 (9.44–11.54)	0.606	0.378
TG (18:1/20:1/22:1)	986.9093 [M + NH_4_]^+^	3.29 (2.50–4.13)	2.51 (1.83–4.16]	0.111	0.128
**TG (16:1/16:1/18:2)**	844.7364 [M + NH_4_]^+^	147.3 (130.4–182.8)	182.8 (156.9–240.3) ^b^	**0.002**	**0.022**
TG (16:1/16:1/17:2)	835.6764 [M + Na]^+^	3.64 (3.20–4.61)	3.53 (3.09–3.89)	0.178	0.169
*TG (16:0/16:0/16:1)*	827.7101 [M + Na]^+^	19.01 (15.59–21.21)	21.55 (17.11–25.06) ^t^	**0.036**	***0.074***
TG (16:1/18:4/18:4)	862.6902 [M + NH_4_]^+^	4.35 (2.97–6.41)	6.37 (3848–9.41) ^a^	**0.014**	**0.047**
TG (16:0/17:2/18:1)	860.7679 [M + NH_4_]^+^	184.9 (156.0–222.3)	190.3 (171.0–220.9)	0.320	0.247
TG (16:0/16:0/17:2)	839.7451 [M + Na]^+^	1.57 (1.37–1.71)	1.47 (1.32–1.64)	0.114	0.131
TG (18:1/18:3/20:0)	935.7928 [M + Na]^+^	3.76 (3.35–4.49)	4.00 (3.24–4.74)	0.639	0.388
TG (18:0/18:1/20:3)	933.7851 [M + Na]^+^	19.46 (17.47–22.81)	18.90 (17.36–23.78)	0.963	0.497
*TG (18:2/18:2/20:0)*	930.8363 [M + NH_4_]^+^	21.21 (18.33–23.40)	17.47(15.95–23.86) ^t^	**0.055**	***0.088***
**TG (18:0/18:1/18:1)**	904.83 [M + NH_4_]^+^	387.1 (333.1–451.4)	341.6 (267.7–385.0) ^a^	**0.015**	**0.049**
*TG (18:0/18:1/18:1)*	906.8364 [M + NH_4_]^+^	81.24 (74.07–95.84)	75.22 (56.50–84.79) ^a^	**0.025**	***0.064***
TG (18:1/18:1/18:1)	902.8144 [M + NH_4_]^+^	956.4 (818.7–1069)	878.3 (810.2–1074)	0.242	0.203
TG (16:0/18:0/18:1)	883.761 [M + Na]^+^	41.08 (33.90–44.66)	42.83 (40.77–45.25)	0.233	0.198
**TG (18:1/18:1/18:2)**	900.7896 [M + NH_4_]^+^	246.9 (224.0–277.0)	225.1 (190.1–236.1) ^a^	**0.005**	**0.032**
*TG (18:1/18:2/18:2)*	898.7739 [M + NH_4_]^+^	120.3 (75.92–128.3)	96.31 (70.34–119.4) ^t^	**0.034**	***0.073***
TG (18:0/18:1/18:2)	902.8052 [M + NH_4_]^+^	260.5 (233.8–281.8)	243.6 (211.4–295.7)	0.246	0.205
**TG (16:0/17:1/20:5)**	882.7478 [M + NH_4_]^+^	8.63 (8.18–9.64)	8.31 (7.60–8.83) ^a^	**0.001**	**0.016**
TG (18:1/20:1/22:3)	982.8773 [M + NH_4_]^+^	2.52 (1.96–3.30)	1.94 (1.43–3.51)	0.119	0.133
*TG(18:1/18:2/20:0)*	930.8455 [M + NH_4_]^+^	78.44 (71.25–95.93)	71.54 (58.23–93.06) ^t^	***0.059***	***0.091***
TG(18:2/20:4/20:4)	944.7669 [M + NH_4_]^+^	39.93 (29.65–52.66)	29.27 (22.13–49.46)	***0.094***	0.118
TG(18:1/20:4/20:4)	946.7742 [M + NH_4_]^+^	8.65 (6.97–10.87)	6.77 (5.28–11.25)	0.101	0.124
TG (20:0/20:0/20:4)	989.8484 [M + Na]^+^	2.27 (1.65–2.85)	1.68 (1.41–2.63)	0.106	0.125
TG (18:2/20:1/20:1)	956.8607 [M + NH_4_]^+^	18.75 (14.96–25.95)	13.52 (11.70–23.93)	0.071	0.102
*TG (18:1/20:2/20:4)*	950.814 [M + NH_4_]^+^	88.26 (75.04–95.26)	74.00 (62.15–90.20) ^a^	**0.022**	***0.059***
TG (18:0/20:3/20:5)	953.7908 [M + Na]^+^	1.89 (1.72–2.32)	1.87 (1.77–2.48)	0.897	0.477
TG (18:2/20:1/20:4)	955.8051 [M + Na]^+^	2.89 (2.46–4.61)	3.50 (2.50–4.72)	0.331	0.250
TG (16:0/18:0/18:0)	880.8213 [M + NH_4_]^+^	119.5 (108.1–154.4)	110.0 (82.96–128.8) ^t^	***0.077***	0.107
TG (18:1/20:0/20:0)	967.8653 [M + Na]^+^	1.69 (1.34–2.62)	1.98 (1.67–3.13)	0.331	0.250
TG (20:1/20:1/20:4)	980.8615 [M + NH_4_]^+^	3.64 (2.69–4.41)	2.63 (2.16–4.60)	0.119	0.133
TG (18:0/20:1/20:4)	959.801 [M + NH_4_]^+^	9.16 (8.57–10.72)	8.57 (7.13–11.61)	0.298	0.235
TG (18:0/20:3/22:0)	991.8645 [M + NH_4_]^+^	2.61 (1.95–3.24)	1.96 (1.69–3.57)	0.153	0.154
TG (20:2/20:4/20:4)	977.7533 [M + NH_4_]^+^	3.58 (2.55–4.31)	2.76 (2.17–4.20)	0.137	0.144
**Sphingomyéline**		178.6 (122.6–220.0)	188.4 (157.9–245.9]	0.480	0.363
**SM (d18:0/12:0)**	651.5340 [M + H]^+^ (isotopic peak)	0.88 (0.52–1.02)	1.04 (0.68–1.54) ^a^	**0.011**	**0.044**
**SM (d18:1/12:0)**	647.5119 [M + H]^+^	3.80 (2.89–5.56)	5.24 (3.99–7.00) ^b^	**0.003**	**0.029**
*SM (18:1/14:0)*	675.5425 [M + H]^+^	72.48 (45.46–98.60)	86.28 (68.91–111.24) ^a^	**0.036**	***0.074***
SM (d18:1/16:0)	725.5552 [M + Na]^+^	41.85 (31.37–52.33)	39.63 (32.05–50.87)	0.907	0.481
SM (d18:1/16:1)	701.5583 [M + H]^+^	13.98 (10.63–20.40)	15.97 (12.92–17.79)	0.574	0.367
SM (d16:1/18:1)	723.5399 [M + Na]^+^	2.76 (2.15–4.12)	2.96 (2.47–3.65)	0.682	0.405
*SM (18:1/20:1)*	779.6015 [M + Na]^+^	3.82 (3.20–4.93)	3.29 (2.50–4.30) ^b^	**0.026**	***0.065***
**SM (d18:1/20:2)**	755.5768 [M + H]^+^	1.49 (1.19–2.17)	1.15 (1.01–1.44) ^a^	**0.010**	**0.042**
SM (18:1/20:1)	757.6205 [M + H]^+^	13.82 (11.32–18.54)	14.51 (12.06–19.27)	1.000	0.506
*SM (d18:1/23:0)*	801.6827 [M + H]^+^	5.97 (3.64–7.23)	7.21 (5.42–9.30) ^a^	**0.048**	***0.081***
SM (d18:1/24:0)	815.6983 [M + H]^+^	7.59 (5.81–10.37)	7.50 (5.59–9.53)	0.433	0.302
**Eicosanoid**					
**10.11-dihydro-20-trihydroxy-leukotriene B4**	385.2364 [M − H]^−^	9.88 (6.00–12.19)	5.66 (4.12–7.09) ^c^	**0.000**	**0.000**
20-Trihydroxy-leukotriene-B4	383.2208 [M − H]^−^	11.22 (4.99–24.61)	9.62 (7.27–15.02)	0.566	0.104
HETE	319.2278 [M − H]^−^	2.32 (1.04–0.90)	2.31 (1.31–4.47)	0.607	0.109
*Leukotriene B4*	335.2227 [M − H]^−^	0.51 (0.28–0.94)	0.32 (0.22–0.60)	0.277	***0.065***
7.8-epoxy-17S-HDHA	357.2051 [M − H]^−^	1.78 (0.80–3.71)	1.60 (0.90–2.47)	0.691	0.119
*15S-HpEDE*	339.2537 [M − H]^−^	1.68 (1.29–2.45)	1.50 (1.27–2.00)	0.292	***0.068***
**11-deoxy-16.16-dimethyl-PGE2**	363.252 [M − H]^−^	52.64 (32.17–70.29)	40.19 (25.54–50.40) ^a^	**0.014**	**0.007**
**9-deoxy-9-methylene-16.16-dimethyl -PGE2**	377.2676 [M − H]^−^	1.38 (0.92–3.30)	0.99 (0.72–1.56)	***0.099***	**0.031**
**PGF2alpha**	353.2314 [M − H]^−^	4.89 (3.26–7.50)	4.12 (2.76–6.73)	0.150	**0.042**
**11-dehydro-2.3-dinor-TXB2**	339.2001 [M − H]^−^	2.98 (1.54–4.17)	3.29 (2.23–4.75)	0.147	**0.041**
**Lyso-PC/PE**		31.55 (25.78–38.13)	29.07 (20.34–40.91)	0.658	0.385
LysoPC (16:0)	454.2921 [M + H]^+^	3.76 (2.85–4.60)	3.21 (2.42–4.48)	***0.099***	0.121
LysoPC (14:0)	468.3079 [M + H]^+^	4.40 (3.16–6.06)	5.39 (3.52–9.12)	0.131	0.141
***LysoPE (16:1)***	452.3133 [M + H]^+^	1.37 (1.03–1.66)	1.65 (1.25–2.02) ^a^	***0.056***	***0.088***
LysoPE (18:1)	480.3079 [M + H]^+^	11.25 (7.84–16.01)	8.82 (6.71–12.77)	***0.079***	0.107
LysoPE (20:5)	500.274 [M + H]^+^	3.34(2.75–4.56)	2.94 (2.13–5.07)	0.198	0.179
LysoPE (20:3)	504.3058 [M + H]^+^	2.96 (2.23–3.56)	3.09 (2.42–4.50)	0.122	***0.058***
*LysoPE (20:4)*	502.2902 [M + H]^+^	3.58 (2.49–4.38)	2.43 (1.74–3.27) ^a^	**0.020**	***0.043***
**LysoPS (22:0)**	580.3535 [M − H]^−^	0.51 (0.32–1.07)	0.43 (0.18–0.86)	0.157	***0.001***
**LysoPG (22:4)**	559.2853 [M − H]^−^	0.70 (0.49–0.89)	0.30 (0.20–0.68) ^c^	**0.000**	***0.019***
**LysoPA (20:0)**	465.3048 [M − H]^−^	4.19 (3.39–4.93)	5.75 (3.75–8.59) ^a^	**0.052**	***0.038***
**Cardiolipine**					
*CL (18:1/18:1/20:4/18:0)*	739.5129 [M − 2H]^−^	2.40 (1.32–3.57)	2.74 (2.04–3.22)	0.419	***0.086***
CL (18:2/20:0/20:0/20:4)	767.5439 [M − 2H]^−^	3.21 (1.45–4.41)	2.87 (1.68–4.54)	0.842	0.134

PC: phosphocholine; PE: phosphoethanolamine; PG: phosphatidylglycerol; PI: phosphoinositol; PS: phosphoserine; SM: sphingomyéline; DG: diacylglycerol; TG: triacylglycerol; CL: cardiolipine. Values are median and [25% and 75% percentile]. Values of *p*-values (assessed by Mann–Whitney *U* test) between “faster” and “slower” growth groups were reported with ^a^, ^b^, ^c^ significantly different; *p* < 0.05, *p* < 0.01 or *p* < 0.001, respectively and *t*, a trend, 0.050 < *p* < 0.10. Lipids in bold font presented a corrected *p*-value < 0.05 ; lipids in bold and in italic font presented a corrected *p*-value between 0.05 and 0.1, using the *post hoc* control of the type I error rate (False discovery Rate procedure), between the two infant groups over the entire W2–W4 lactation period.

**Table 4:** Predictive ability of tentative lipid and fatty acid biomarkers on infant’ growth (defined based on the difference between discharge and birth weight *Z*-score).

	Infant’ Weight Growth between Birth and Discharge (SD) (*p*-Value Corrected–MLR)
***Fatty Acids (Targeted Analysis)***	
**12:00**	**0.0043**
**14:00**	**0.0005**
*SAT*	0.2429
***MCSAT***	**0.0065**
**18:1*_n_*_−9_**	**0.0101**
***MUFA***	**0.0059**
***MUFA/SFA***	**0.0509**
**18:1*_n_*_−9 et *n*−7_**	**0.0104**
***cis*** **20:5*_n_*_−3_ (EPA)**	**0.0606**
*cis* 22:5*_n_*_−3_ (DPA)	0.2651
*cis* 22:6*_n_*_−3_ (DHA)	0.1081
*Total* n*-3 PUFA*	0.3299
*Unsaturated/saturated fatty acid*	0.3179
n-*6/*n-*3 PUFA*	0.5089
*LA/ALA*	0.674
*AA/DHA*	0.3199
***Fatty acids (lipidomics analysis)***	
3-Hydroxyadipic acid	0.3109
*N*-formylmaleamic acid	0.3362
Dodecatetraenedioic acid	0.1633
Linderic acid	0.1977
**alpha-hydroxy lauric acid**	**0.0993**
3-oxo-4-pentenoic acid	0.2546
Dehydrocholic acid	0.3842
7*R*,9,14*R*-trimethyl-2*E*,4*E*,8*E*,10*E*-hexadecatetraenoic acid	0.3054
***Ceramide***	
Cer(d18:1/24:0)	0.2395
***Sphingomyeline***	
**SM (d18:0/12:0)**	**0.0919**
SM (d18:1/12:0)	0.4359
SM (18:1/14:0)	0.6205
SM (18:1/20:1)	0.3694
SM (d18:1/20:2)	0.2828
SM (d18:1/23:0)	0.8853
SM (d18:1/24:0)	0.1673
***Glucosy/Galactosyl-Ceramide***	
GlucosylCeramide (d18:1/18:0)	0.5277
***Phosphatidylcholine***	
**PC (18:0/18:1)**	**0.0445**
PC (20:0/20:2)	0.3251
PC (16:1/18:2)	0.7613
PC (18:1/18:1)	0.6294
PC (18:0/18:2)	0.8524
PC (16:0/20:3)	0.3092
PC (18:0/20:3)	0.4509
PC (20:1/20:4)	0.8255
PC (20:3/22:6)	0.2408
PC (16:0/22:6)	0.6206
***PC-plasmalogen***	
PC (P-18:0/18:0)	0.6294
***Phosphatidylethanolamine***	
PE (16:0/16:1)	0.7887
PE (16:1/20:0)	0.958
PE (16:0/20:2)	0.3531
PE (18:0/20:4)	0.4997
PE (22:0/20:3)	0.9017
PE (18:2/18:2)	0.8205
PE (20:0/18:1)	0.4662
PE (20:4/20:0)	0.7422
**PE (20:3/22:6)**	**0.0497**
PE (18:0/20:3)	0.1121
***PE-plasmalogen***	
*PE (O-18:0/20:5)*	0.0331
***Phosphatidylglycerol***	
PG (P-16:0/22:4)	0.846
PG (18:2/20:5)	0.3118
PG (22:2/22:6)	0.2912
***Diacylglyceride***	
DG (20:3:/20:0)	0.2475
***Triglyceride***	
TG (14:0/16:0/16:0)	0.1971
**TG (14:0/14:1/19:1)**	0.5987
TG (16:0/16:1/16:1)	0.9338
TG (14:0/16:0/16:0)	0.7951
**TG (16:0/17:1/18:1)**	**0.0122**
TG (16:1/16:1/18:2)	0.6581
TG (16:0/16:0/16:1)	0.9685
**TG (16:1/18:4/18:4)**	**0.0034**
TG (18:2/18:2/20:0)	0.8228
**TG (18:0/18:1/18:1)**	**0.066**
TG (18:1/18:1/18:2)	0.4819
TG (18:1/18:2/18:2)	0.4212
TG (16:0/17:1/20:5)	0.9923
TG (18:1/18:2/20:0)	0.3693
TG (18:1/20:2/20:4)	0.5425
***Eicosanoïd***	
10,11-dihydro-20-trihydroxy-leukotriene B4	0.909
Leukotriene B4	0.3869
**15S-HpEDE**	**0.0042**
**11-deoxy-16,16-dimethyl-PGE2**	**0.0017**
**9-deoxy-9-methylene-16,16-dimethyl-PGE2**	**0.0075**
PGF2alpha	0.2057
**11-dehydro-2,3-dinor-TXB2**	0.5135
***Lyso PS/PG***	
**LysoPS (22:0)**	**0.0849**
**LysoPG (22:4)**	**0.0741**
LysoPA (20:0)	0.9564
***Cardiolipine***	
CL (18:1/18:1/20:4/18:0)	0.3702

Values of *p*-values were calculated using multiple linear regression analysis, by taking into account several confounding factors (infant’s birth weight, mother’s BMI, gestational age, complementary parenteral and enteral nutrition with the protein, lipid and energy intakes, duration of parenteral feeding and ventilation, and length of hospital stay). Statistical significance was set to a confidence level of *p* < 0.10 (*p*-values in bold font).

## Supplementary Materials

The following are available online at http://www.mdpi.com/2072-6643/10/2/164/s1, Figure S1: Multi-group PLS-DA score plots based on the LC-ESI^−^-HRMS profiles obtained on human preterm milk; Figure S2: AoV-PLS and LDA models, based on the LC-ESI^−^-HRMS profiles of human preterm milk, on the factor weight *Z*-score (discharge-birth); Table S1: Concentration levels of total fatty acids expressed as % of total identified FA, measured in human preterm milk at W2, W3, W4 of lactation and provided to newborns with “faster” or “slower” growth; Table S2: Abundance (10^6^) of annotated discriminant lipids measured in breast milk at week 2 (W2), W3, W4 of lactation provided to preterm infants with fast or slow growth.

## Abbreviations

AA	arachidonic acid
ALNA	alpha-Linolenic acid
AoV-PLS	analysis of variance combined to partial least squares regression
AUC	area under the curve
CL	cardiolipine
DG	diacylglycerol
DGLA	dihomo-gamma-linolenic acid
DHA	docosahexanoic acid
GA	gestational age
GLNA	gamma-linolenic acid
EPA	eicosapentaenoic acid
ESI	electrospray ionization
FDR	false discovery rate
LA	linoleic acid
LC-HR-MS	liquid-chromatography–high-resolution-mass-spectrometry
LC-PUFA	long-chain PUFA
MG PLS-DA	multi-group partial least squares discriminant analysis
MLR	multiple linear regression
MCSAT	medium-chain saturated fatty acids
MUFA	mono-unsaturated fatty acids
PC	phosphocholine
PE	phsosphethanolamine
PG	phosphatidylglycerol
PI	phosphoinositol
PS	phosphoserine
PUFA	polyunsaturated fatty acid
SAT	saturated fatty acids
ROC	receiver operating-characteristic
SD	standard deviation
SM	sphingomyéline
TG	triacylglycerol
W4M	workflow4metabolomics^®^
AA	arachidonic acid
ALNA	alpha-linolenic acid
AoV-PLS	analysis of variance combined to partial least squares
AUC	area under the curve
CL	cardiolipine
DG	diacylglycerol
DGLA	dihomo-gamma-linolenic acid
DHA	docosahexanoic acid
GA	gestational age
GLNA	gamma-linolenic acid
EPA	eicosapentaenoic acid
ESI	electrospray ionization
FDR	false discovery rate
LA	linoleic acid
LC-HR-MS	liquid-chromatography–high-resolution-mass-spectrometry
LC-PUFA	long-chain PUFA
MCSAT	medium chain-saturated fatty acid
MG PLS-DA	multi-group partial least squares-discriminant analysis
MLR	multiple linear regression
MUFA	mono-unsaturated fatty acids
PC	phosphocholine
PE	phosphoethanolamine
PG	phosphatidylglycerol
PI	phosphoinositol
PS	phosphoserine
PUFA	polyunsaturated fatty acid
SAT	saturated fatty acids
ROC	receiver operating-characteristic
SD	standard deviation
SM	sphingomyéline
TG	triacylg lycerol
W4M	workflow4metabolomics^®^
